# Prevalence of Insomnia Among Residents of Tokyo and Osaka After the Great East Japan Earthquake: A Prospective Study

**DOI:** 10.2196/ijmr.2485

**Published:** 2013-01-18

**Authors:** Hiroaki Sugiura, Manabu Akahane, Yasushi Ohkusa, Nobuhiko Okabe, Tomomi Sano, Noriko Jojima, Harumi Bando, Tomoaki Imamura

**Affiliations:** ^1^Health Management and PolicyDepartment of Public HealthNara Medical University School of MedicineKashiharaJapan; ^2^National Institute of Infectious DiseasesInfectious Disease Surveillance CenterTokyoJapan; ^3^Faculty of NursingNara Medical University School of MedicineKashiharaJapan

**Keywords:** insomnia, Web-based survey, population surveillance, disaster, nuclear accidents, earthquakes

## Abstract

**Background:**

The Great East Japan Earthquake occurred on March 11, 2011. Tokyo and Osaka, which are located 375 km and 750 km, respectively, from the epicenter, experienced tremors of 5.0 lower and 3.0 seismic intensity on the Japan Meteorological Agency scale. The Great East Japan Earthquake was the fourth largest earthquake in the world and was accompanied by a radioactive leak at a nuclear power plant and a tsunami. In the aftermath of a disaster, some affected individuals presented to mental health facilities with acute stress disorder (ASD) and/or post-traumatic stress disorder (PTSD). However, few studies have addressed mental stress problems other than ASD or PTSD among the general public immediately after a disaster. Further, the effects of such a disaster on residents living at considerable distances from the most severely affected area have not been examined.

**Objective:**

This study aimed to prospectively analyze the effect of a major earthquake on the prevalence of insomnia among residents of Tokyo and Osaka.

**Methods:**

A prospective online questionnaire study was conducted in Tokyo and Osaka from January 20 to April 30, 2011. An Internet-based questionnaire, intended to be completed daily for a period of 101 days, was used to collect the data. All of the study participants lived in Tokyo or Osaka and were Consumers’ Co-operative Union (CO-OP) members who used an Internet-based food-ordering system. The presence or absence of insomnia was determined before and after the earthquake. These data were compared after stratification for the region and participants’ age. Multivariate analyses were conducted using logistic regression and a generalized estimating equation. This study was conducted with the assistance of the Japanese CO-OP.

**Results:**

The prevalence of insomnia among adults and minors in Tokyo and adults in Osaka increased significantly after the earthquake. No such increase was observed among minors in Osaka. The overall adjusted odds ratios for the risk of insomnia post-earthquake versus pre-earthquake were 1.998 (95% CI 1.571–2.542) for Tokyo, 1.558 (95% CI 1.106–2.196) for Osaka, and 1.842 (95% CI,1.514–2.242) for both areas combined.

**Conclusions:**

The prevalence of insomnia increased even in regions that were at a considerable distance from the epicenter. Both adults and minors in Tokyo, where the seismic intensity was greater, experienced stress after the earthquake. In Osaka, where the earthquake impact was milder, disturbing video images may have exacerbated insomnia among adults.

## Introduction

On March 11, 2011, the Japanese islands sustained a 9.0-magnitude earthquake. Unlike previous major earthquakes in Japan [1,2], this earthquake was followed by a tsunami that devastated the affected areas [3]. More than 20,000 individuals were recorded as dead or missing. The tsunami also caused extensive damage to the Fukushima Daiichi nuclear power plant, resulting in a level 7 nuclear accident [4,5]. This induced considerable anxiety among residents living near the nuclear power plant and among people living as far away as the Tokyo metropolitan area [6]. Images of the tsunami and scenes of the nuclear accident were shown repeatedly on television and the Internet.

In the aftermath of a disaster, people may experience not only physical disorders but also acute stress disorder (ASD), which can persist for up to 4 weeks. Furthermore, chronic post-traumatic stress disorder (PTSD) is common among individuals who have faced such situations [7]. Studies of disaster-related mental disorders typically include an assessment of the prevalence of PTSD, follow-up of patients diagnosed with ASD [8], and a comparison of the numbers of new and previous cases of PTSD in a given area. However, because these studies are usually planned after a disaster, pre-disaster prevalence must be determined retrospectively. A recollection of previous insomnia is likely to be less accurate than the prospective reporting of current symptoms of insomnia, especially during the traumatic aftermath of a disaster.

The current study made use of a daily health survey that was administered to 3128 participants in Tokyo and 1925 participants in Osaka (Table 1) from January 20 to April 30, 2011. One question on the survey specifically asked about the presence or absence of insomnia. Because the Great East Japan Earthquake occurred during the course of this survey, this was a rare opportunity to prospectively assess the impact of an earthquake on the prevalence of insomnia among residents of Tokyo and Osaka.

**Table 1:** Number of participants according to sex and age group.

	Tokyo N (male/female)	Osaka N (male/female)
Adults (≥20 years of age)	2073 (999/1074)	1182 (564/618)
Minors (<20 years of age)	1055 (575/480)	743 (373/370)

## Methods

### Study Period and Locations

**Figure 1:**
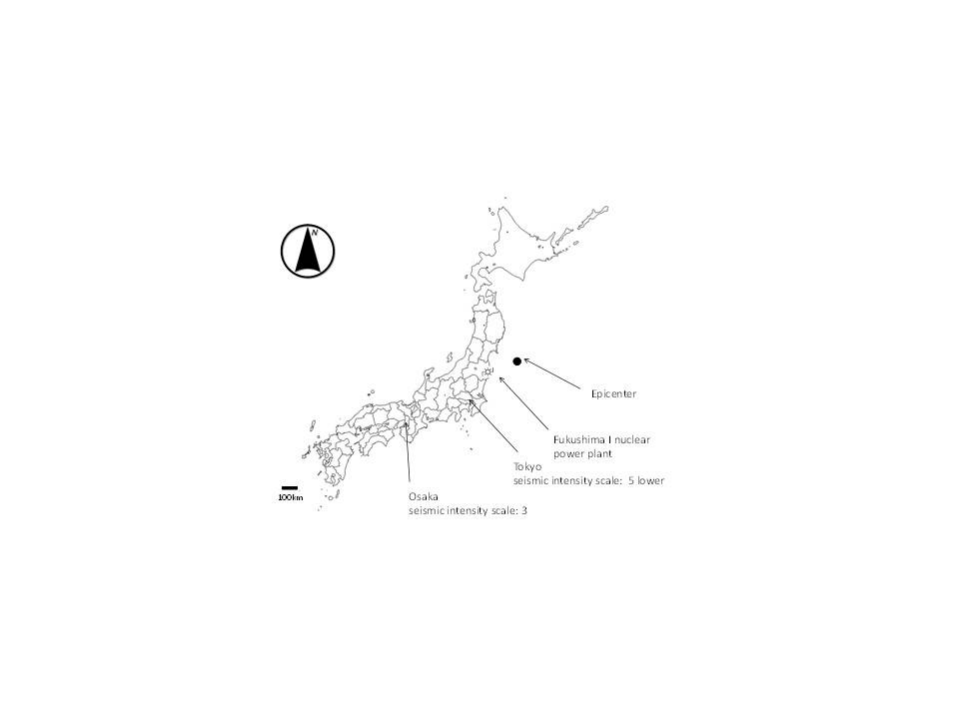
Map of the locations relevant to this study.

This survey began on January 20, 2011 and continued for 101 days until April 30, 2011. The questionnaire collected data related to the individual’s health status on the day of the survey, and participants were instructed to complete the survey every day for the duration of the study period. The survey was conducted via an Internet-based questionnaire among residents of the Tokyo metropolitan area and Osaka, the largest city in western Japan. Tokyo is located approximately 375 km from the epicenter of the earthquake (N 38°06′ E 142°51′) and approximately 200 km from the Fukushima Daiichi nuclear power plant (N 37°42′ E 141°03′). The seismic intensity of the main shock in the center of Tokyo, as recorded by the Japan Meteorological Agency (JMA), was 5.0 Lower on the JMA scale [9]. The JMA scale is comprised of 5 phases from 1 to 5. Grades 5 and 6 are further classified into 2 subcategories: upper and lower. During an earthquake with an intensity of 5.0 Lower, people may find it difficult to move around, but major destruction is generally not expected. In contrast, many people find it hard to move during earthquakes with an intensity of 5.0 Upper [9]. Shinjuku Ward, where the offices of the Tokyo Metropolitan Government are located, was subsequently hit by 10 aftershocks that continued until April 16, 2011. The seismic intensity of the aftershocks was ≥3.0, strong enough to be felt by most people inside buildings [9]. Osaka, the other area investigated in the survey, is situated 750 km from the epicenter of the earthquake. The seismic intensity of the main shock was recorded as 3.0 in the offices of the Osaka Prefectural Government. Osaka did not receive any aftershocks with a seismic intensity ≥3.0 (Figure 1).

### Participants

This study was conducted with the assistance of the Japanese Consumers’ Co-operative Union (CO-OP). All respondents who completed the questionnaire lived in Tokyo or Osaka and resided in households that included CO-OP members who placed food orders via the CO-OP website.

### Survey Method

This study was conducted with the approval of the Ethics Committee of Nara Medical University (authorization code: 220). The general health condition of the participants, including their sleeping patterns, was investigated using an Internet-based questionnaire. The original aims of this survey were to determine the impact of biological factors, such as infectious diseases, and abiotic factors, such as climate, on the physical condition of residents during the study period. The survey method and data processing methods were described in detail in our previous study [10].

### Registration Method

Respondents were recruited through a banner advertisement on the CO-OP’s website. Each participant was rewarded with 500 yen (US $1=91.15 yen on the first day of the survey) upon registration for participating in the survey. No remuneration, in the form of cash, was given for providing answers on a daily basis.

### Daily Survey Method

The original research plan was to send a reminder email to all the respondents on each day of the survey that would direct them to the website where they could provide their responses. The email was distributed as planned until day 50 of the survey. The Great East Japan Earthquake occurred on day 51 of the survey. The reminder emails were discontinued, as it was decided that the participants, who were recovering from the disaster, should not be burdened. Further responses were left to the participants’ discretion during a hiatus period from March 14 to April 5, 2011, when the reminders were reinstituted. After the earthquake, respondents were able to submit descriptions of their physical condition by voluntarily visiting the website.

The daily survey procedure was designed to be simple. After confirming the everyday health condition of the family, participants were asked to access the survey website and answer several questions. The first question asked whether any family member was in poor health. If the participant answered “no”, they were excluded from the survey. If the participant answered “yes”, they were asked to answer additional “yes” or “no” questions on 19 symptoms; these questions pertained to the individual filling out the questionnaire as well as each member of his or her family [10]. The presence or absence of insomnia was prospectively investigated for 50 days before and 51 days after the Great East Japan Earthquake (including the day of the earthquake).

### Statistical Analysis

In both surveyed areas, the prevalence of insomnia was calculated on a daily basis (the number of people reporting symptoms of insomnia divided by the number of responses per day) among people aged <20 years and those aged ≥20 years. Using a chi-square test, the presence or absence of insomnia before and after the earthquake was investigated for any correlation with region or participant age. A multivariate analysis was carried out using logistic regression analysis and a generalized estimating equation. The presence or absence of insomnia was the dependent variable. The independent variables included insomnia occurring after the earthquake, sex, age, region of each participant, the status of reminder emails (sent or not), and the incidence of pollinosis, which plagued approximately 30% of adults in those urban areas during the spring [11]. The statistical analyses were carried out using SPSS version 19.0 (IBM, Chicago, IL, USA).

## Results

### Response Rate

The mean (SD) daily response rate during the period when reminder emails were sent was 64.17% (5.78%) for Tokyo and 68.31% (5.18%) for Osaka. The response rate did not decline significantly over the course of the study. The response rate during the period when no reminder emails were sent (March 14 to April 5, 2011) was 24.47% (12.97%) for Tokyo and 27.82% (13.55%) for Osaka.

### Daily Prevalence of Insomnia

**Figure 2:**
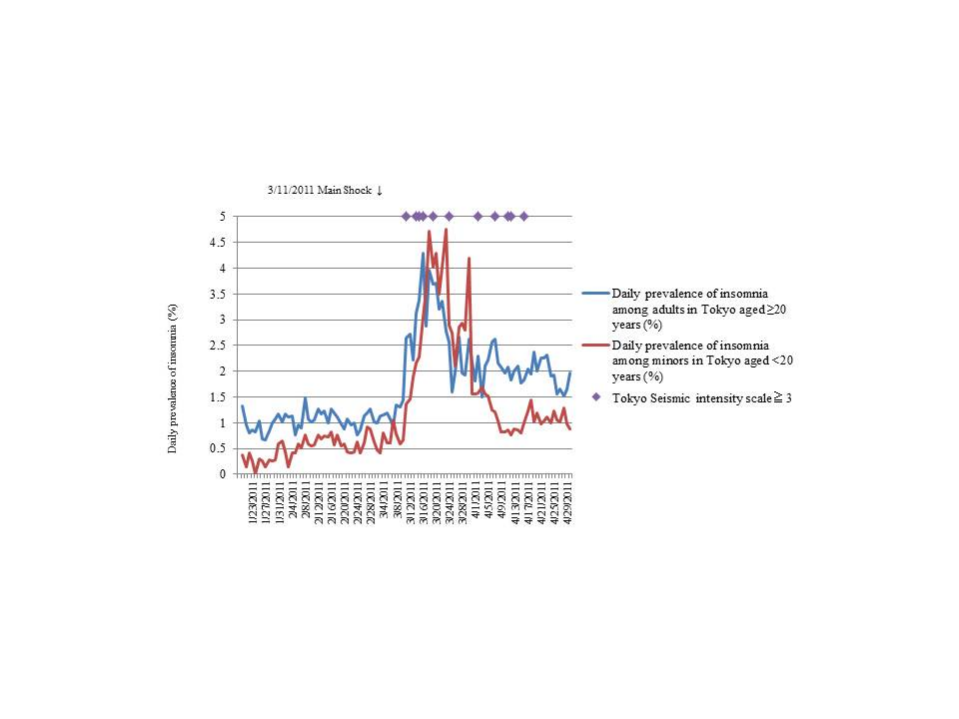
Prevalence of insomnia in Tokyo. The prevalence of insomnia increased after the earthquake for both adults and minors in Tokyo.

**Figure 3:**
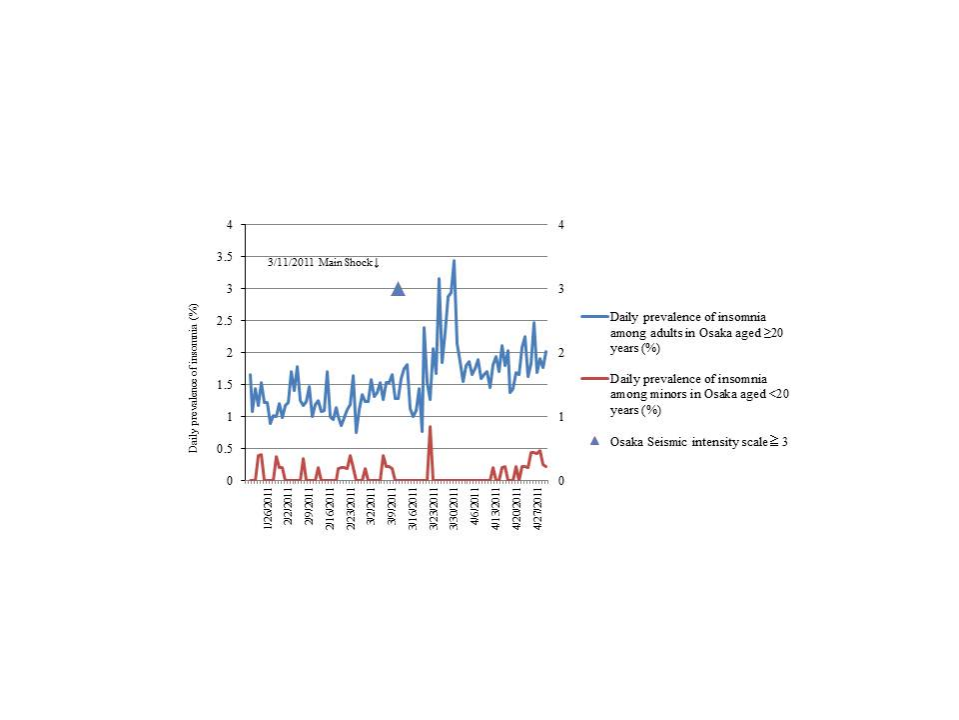
Prevalence of insomnia in Osaka. The prevalence of insomnia among adults increased after the earthquake. The prevalence of insomnia among minors remained approximately the same as that before the earthquake.

Figures 2 and 3 illustrate the daily prevalence of insomnia in Tokyo and Osaka, respectively, according to age. The figures also indicate the dates of the main earthquake and the aftershocks with seismic intensity ≥3.0. Before the earthquake, the average daily prevalence of insomnia in Tokyo was 1.05% (0.18%) for adults (age ≥20 years) and 0.53% (0.22%) for minors (age <20 years); after the earthquake, this value increased to 2.35% (0.65%) for adults and 1.90% (1.17%) for minors. The maximum seismic intensity of the main earthquake was 5.0 Lower in Tokyo (Figure 2).

Before the earthquake, the average daily prevalence of insomnia in Osaka was 1.25% (0.25%) for adults and 0.092% (0.14%) for minors; after the earthquake, this value increased to 1.83% (0.51%) for adults but remained approximately the same at 0.089% (0.17%) for minors. The maximum seismic intensity of the main earthquake was 3.0 in Osaka (Figure 3).

A chi-square test was conducted to analyze the data according to region and age group. There was a significant increase in the number Tokyo residents who reported symptoms of insomnia after the earthquake (*P*<.001 for both adults and minors) compared with that before the earthquake. The same findings were reported for adults in Osaka after the earthquake (*P*<.001). No significant difference was observed among minors in Osaka (Table 2). We conducted a similar chi-square test that excluded the period during which no reminder emails were sent and similar results were obtained.

**Table 2:** Chi-square analysis according to sex and age.

Region		Chi-square value	Degrees of freedom	*P*	Odds ratio	95% CI
Tokyo	Adults	246.63	1	<.001	2.107	1.916–2.317
	Minors	128.52	1	<.001	2.763	2.301–3.319
Osaka	Adults	34.65	1	<.001	1.438	1.273–1.623
	Minors	0.087	1	.77	1.096	0.595–2.020

### Analysis of Factors Associated with the Prevalence of Insomnia

Multivariate analysis was conducted to determine the odds ratios for insomnia (Table 3). The adjusted odds ratios for insomnia after versus before the earthquake were 1.998 (95% CI 1.571–2.542, *P*<.001) for Tokyo, 1.558 (95% CI 1.106–2.196, *P*=.011) for Osaka, and 1.842 (95% CI1.514–2.242, *P*<.001) for the 2 areas combined. Table 3 presents the factors analyzed in this study and their associations with the prevalence of insomnia.

**Table 3:** Multivariate analysis of factors associated with the prevalence of insomnia.

			Odds ratio	*P*	95% CI
**Predictor for sleeplessness** ^**a**^					
	Post-earthquake vs pre-earthquake		1.842	<.001	1.514–2.242
	Age ≥20 years vs age <20 years		2.246	.027	1.095–4.605
	Female vs male		1.510	.109	0.912–2.501
	Presence vs absence of pollinosis		2.334	.001	1.437–3.791
	Tokyo vs. Osaka		1.404	.187	0.848–2.323
	No reminder email vs reminder email		1.303	.016	1.050–1.617
**Predictor of sleeplessness**					
	**Tokyo**				
		Post-earthquake vs pre-earthquake	1.998	<.001	1.571–2.542
		Age ≥20 years vs age <20 years	1.378	.421	0.631–3.010
		Female vs male	1.670	.903	0.90–3.087
		Presence vs absence of pollinosis	2.437	.005	1.317–4.509
		No reminder email vs reminder email	1.435	.004	1.121–1.838
	**Osaka**				
		Post-earthquake vs pre-earthquake	1.558	.011	1.106–2.196
		Age ≥20 years vs age <20 years	13.987	<.001	6.408–30.530
		Female vs male	1.285	.554	0.554–2.983
		Presence vs absence of pollinosis	2.193	.047	1.012–4.751
		No reminder email vs reminder email	1.005	.983	0.658–1.535

^a^ values are total counts from Tokyo and Osaka

## Discussion

### Overall

This study examined the prevalence of insomnia among residents in areas that were at different distances from the epicenter of the Great East Japan Earthquake. This is a unique study in that it analyzes the effect of a great earthquake on the rates of insomnia and includes a pre-event baseline in the same group.

### Great East Japan Earthquake and Its Impact

Major earthquakes have been common throughout the Asia-Pacific region over the past 2 decades [12,13], with more major earthquakes occurring in Japan than in any other country. In recent decades, 4 particularly large earthquakes have hit Japan, including the Great Hanshin Earthquake of 1995, which hit the Osaka region [1,2,14,15]. The Great East Japan Earthquake was the fourth largest earthquake in the world and was accompanied by 2 major events that could have occurred only in a modern society. First, the earthquake caused a radioactive leak at a nuclear power plant. Second, video images of the ensuing tsunami were recorded, and the footage was shown repeatedly on television; they were also available on the Internet. These images had a profound psychological impact on viewers. In the aftermath of a disaster, affected individuals may present to mental health facilities with ASD and/or PTSD [16-19]. However, few studies have addressed mental stress problems other than ASD or PTSD among the general public immediately after a disaster. Although ASD and PTSD tend to draw greater research attention in studies related to a major disaster, the effects of such a disaster on residents living at considerable distances from the most severely affected area have not been examined. This study revealed an increase in the prevalence of insomnia among the general public immediately after the occurrence of a major earthquake. To our knowledge, this is the first study conducted in Japan that presents longitudinal data on the persistence of insomnia in 2 age groups.

### Daily Prevalence of Insomnia in Tokyo and Osaka

The daily prevalence of insomnia increased among both adults and minors in Tokyo after the Great East Japan Earthquake. Although the daily prevalence of insomnia increased among adults in Osaka, a similar increase was not observed among minors. The adjusted odds ratios for insomnia after versus before the earthquake were 1.998 (95% CI 1.571–2.542) for Tokyo, 1.558 (95% CI 1.106–2.196) for Osaka, and 1.842 (95% CI 1.514–2.242) for the 2 areas combined. These results demonstrate an increased prevalence of insomnia among residents in regions located at considerable distances from the immediate zone of the disaster. In Tokyo, where there was no observable infrastructure damage due to the tsunami, 7 people died as a result of the initial tremor. In addition, many people in Tokyo experienced considerable psychological strain for a prolonged period. Many commuters were stranded because of interrupted transportation services, and there was a high risk of radioactive contamination associated with the nuclear accident. The increased prevalence of insomnia among minors in Tokyo, who are generally less susceptible to stress induced by indirect sources such as media coverage, may be attributable to the effects of the aftershocks. In contrast, the seismic intensity of the main shock in Osaka was 3.0; therefore, direct feelings of fear were likely to be less common, and there was an absence of palpable aftershocks. The prevalence of insomnia among minors in Osaka following the earthquake was not increased, which can be explained by the residents’ exposure to fewer direct and local effects. However, an increased number of adults in Osaka reported insomnia. This may have stemmed from exposure to information reported by the media. Other possible causes of insomnia among these adults include anxiety about their future and memories of the disaster caused by the Great Hanshin Earthquake of 1995.

### Questionnaire Survey and Its Advantages

A Web-based questionnaire survey was used in the current study because more data are acquired with Internet-based epidemiological surveys than with conventional, paper-based surveys [20,21]. This method was effective in targeting general residents and enabling the acquisition of information from people with medical complaints deemed very mild to warrant a visit to a medical facility. In addition, this survey method was successful because the participants were required to respond only to simple questions regarding the presence or absence of symptoms, thus, the input burden was low. Although a meta-analysis of 68 studies [22] indicated that the normal response rate to Internet-based surveys is low (39.6%), the daily response rate for this study during the period when reminder emails were sent was 64.17% (5.78%) for Tokyo and 68.31% (5.18%) for Osaka. The survey questions were not specifically designed to detect post-disaster psychological conditions, and insomnia was only 1 of several conditions investigated. Participants’ responses were limited to the presence or absence of insomnia, and there was no attempt to determine the severity of the condition. Because insomnia was investigated as only 1 of several conditions, participants were unaware that their responses would be used in a study on post-disaster stress, even after the earthquake struck. It is possible, therefore, that the participants were less inclined to answer “yes” to the question about any experience of insomnia symptoms. This possibility is supported by the fact that the average daily prevalence of insomnia among adults before the earthquake was 1.1% in Tokyo and 1.3% in Osaka; these rates are lower than the values reported by an earlier survey on the prevalence of insomnia among Japanese adults [23].

### Limitations

Immediately after the earthquake struck, an ethical decision was made to refrain from sending reminder emails. Therefore, the response rate was low during this period. However, no significant difference in the daily prevalence of insomnia correlated with the use of these reminder emails in either Tokyo or Osaka. The chi-square test results were similar between analysis including and excluding this time period. Although the reminder emails were included in the logistic regression analysis as an independent variable, the presence or absence of the reminder emails inevitably remains a limitation of this study and a potential source of bias. However, we believe that this factor had a negligible effect on the results.

### Conclusions

This study examined the prevalence of insomnia among residents in areas distant from the epicenter of the Great East Japan Earthquake. In Tokyo, where the seismic intensity was higher, both adults and minors experienced increased rates of insomnia as a direct result of the earthquake and its aftershocks. Further, mental stress induced by information broadcast by the media may have influenced the prevalence of insomnia. In Osaka, where the seismic intensity was lower, only adults exhibited an increased prevalence of insomnia. Health care practitioners should be aware that individuals might experience mental stress, including insomnia, even in areas distant from those that are directly affected by a natural disaster.

## Abbreviations

ASD: acute stress disorder
CO-OP: Consumers’ Co-operative Union
JMA: Japan Meteorological Agency
PTSD: post-traumatic stress disorder
